# Supplementary material for: Optimal regulation of blood glucose level in Type I diabetes using insulin and glucagon
Source: PLoS One. 2019 Mar 20;14(3):e0213665. doi: 10.1371/journal.pone.0213665 (PMC6426249; doi:10.1371/journal.pone.0213665)
Supplement: S1 Appendix — The nonlinear equations of the GIGM model are given in Eqs (S1)-(S9). The parameters are given in S1 Table. The equations are given in Eq (S10) and the basal steady states are given in Eq (S11). The basal values are given in S2 Table. The references [67–69] are appeared here. (PDF) [file pone.0213665.s001.pdf]

S1 Appendix: GIGM Model and Parameters

Overview of GIGM Model with Type I Diabetics

We consider the model in [3,4] which is a system of nonlinear differential equations (ODEs). In all equations, t is the physical time (in min), all subscripts b denotes basal state, and all of the parameters are given in the S1 Table. The system of nonlinear differential equations are given below:

Glucose Subsystem:

$$\dot{G}_p(t) = EGP(t) + Ra(t) - U_{ii} - E(t) - k_1 G_p(t) + k_2 G_t(t), \qquad G_p(0) = G_{pb} \tag{S1a}$$

$$\dot{G}_t(t) = -U_{id}(t) + k_1 G_p(t) - k_2 G_t(t), \qquad G_t(0) = G_{tb} \tag{S1b}$$

$$G(t) = \frac{G_p}{V_G}, \qquad G(0) = G_b \tag{S1c}$$

Here G_p (in mg/kg) is the mass of plasma glucose; G_t (in mg/kg) is the mass of tissue glucose; G (in mg/dL) is plasma glucose concentration and V_g (in dL/kg) is the distribution volume of glucose; EGP is the endogenous glucose production (in mg/kg/min); Ra (in mg/kg/min) is the rate of glucose appearance in plasma; U_{ii} (in mg/kg/min) and U_{id} (in mg/kg/min) are insulin-independent and insulin-dependent glucose utilizations, respectively. Also k_1 and k_2 are the parameters.

Insulin Subsystem:

$$\dot{I}_p(t) = -(m_2 + m_4) I_p(t) + m_1 I_l(t) + R_{ia}(t), \qquad I_p(0) = I_{pb} \tag{S2a}$$

$$\dot{I}_l(t) = -(m_1 + m_3) I_l(t) + m_2 I_p(t), \qquad I_l(0) = I_{lb} \tag{S2b}$$

$$I(t) = \frac{I_p(t)}{V_I}, \qquad I(0) = I_b \tag{S2c}$$

Here I_l (in pmol/kg) is the mass of liver insulin; I_p (in pmol/kg) is the mass of tissue insulin; I (in pmol/L) is the plasma insulin concentration; V_I (in L/kg) is the distribution volume of insulin; R_{ia} (in pmol/kg/min) is the rate of appearance of insulin in plasma; m_1, m_2, m_3 and m_4 are the parameters.

Glucose rate of appearance:

$$Q_{sto}(t) = Q_{sto1}(t) + Q_{sto2}(t), \qquad G_{sto}(0) = 0 \tag{S3a}$$

$$\dot{Q}_{sto1}(t) = -k_{gri} Q_{sto1}(t) + D\delta(t - \tau_D), \qquad Q_{sto1}(0) = 0 \tag{S3b}$$

$$\dot{Q}_{sto2}(t) = -k_{empt}(Q_{sto})(t) Q_{sto2}(t) + k_{gri} Q_{sto1}(t), \qquad Q_{sto2}(0) = 0 \tag{S3c}$$

$$\dot{Q}_{gut}(t) = -k_{abs} Q_{gut}(t) + k_{empt} Q_{sto}(t) Q_{sto2}(t), \qquad Q_{gut}(0) = 0 \tag{S3d}$$

$$Ra(t) = \frac{f.k_{abs}.Q_{gut}(t)}{BW}, \qquad Ra(0) = 0 \tag{S3e}$$

$$k_{empt}(Q_{sto}) = k_{\min} + \frac{k_{\max} - k_{\min}}{2}. \tag{S3f}$$

$$\{\tanh[\alpha(Q_{sto} - b.D)] - \tanh[\beta(Q_{sto} - c.D)] + 2\} \tag{S3g}$$

Here Q_{sto} (in mg) is the amount of glucose in the stomach, Q_{sto1} (in mg) is the amount of liquid glucose in the stomach, Q_{sto2} (in mg) is the amount of solid glucose in the stomach, Q_{gut} (in mg) is the glucose mass in the intestine; D (in mg) is the amount of ingested glucose at time

τ_D; BW (in kg) is body weight; k_{empt} is the rate constant of the gastric emptying; K_{gri}, k_{abs}, k_{max}, k_{min}, f, α, β are the parameters.

Endogenous glucose production:

$$EGP(t) = k_{p1} - k_{p2}G_p(t) - k_{p3}X^L(t) + \xi X^H(t), \qquad EGP(0) = EGP_b \tag{S4a}$$

$$\dot{I}'(t) = -k_i\left[I'(t) - I(t)\right], \qquad I'(0) = I_b \tag{S4b}$$

$$\dot{X}^L(t) = -k_i\left[X^L(t) - I'(t)\right], \qquad X^L(0) = I_b \tag{S4c}$$

$$\dot{X}^H(t) = -k_H X^H(t) + k_H \times \max\left[H(t) - H_b, 0\right], \qquad X^H(0) = 0 \tag{S4d}$$

Here X^L (in) is delayed insulin action on EGP; X^H is delayed glucagon action on EGP; I' is delayed insulin in compartment 1; k_{p1}, k_{p2}, k_{p3}, ξ, k_i, k_H are the parameters.

Glucose utilization:

$$U_{ii}(t) = F_{cns} \tag{S5a}$$

$$U_{id}(t) = \frac{[V_{m0} + V_{mx}.X(t)]G_t(t)}{K_{m0} + G_t(t)} \tag{S5b}$$

$$\dot{X}(t) = -p_{2U}X(t) + p_{2U}[I(t) - I_b], \qquad X(0) = 0 \tag{S5c}$$

Here U_{ii} (in mg/kg/min) and U_{id} (in mg/kg/min) are insulin-independent and insulin-dependent glucose utilization; X (in pmol/L) is insulin in interstitial fluid; F_{cns}, V_{m0}, K_{m0}, p_{2U} are the parameters.

Renal excretion:

$$E(t) = \begin{cases} k_{e1}[G_p(t) - k_{e2}] & \text{if } G_p(t) > k_{e2} \\ 0 & \text{if } G_p(t) \leq k_{e2} \end{cases} \tag{S6}$$

Here $E(t)$ (in mg/kg/min) is the glucose renal exertion; k_{e1} is the parameter.

Glucagon kinetics and secretion:

$$\dot{H}(t) = -nH(t) + SR_H(t) + Ra_H(t), \qquad H(0) = H_b \tag{S7a}$$

$$SR_H(t) = SR_H^s(t) + SR_H^d(t), \tag{S7b}$$

$$\dot{SR}_H^s(t) = -\rho\left[SR_H^s(t) - \max\left(\frac{\sigma[G_{th} - G(t)]}{\max(I(t) - I_{th}, 0) + 1} + SR_H^b, 0\right)\right], \qquad SR_H^s(0) = nH_b \tag{S7c}$$

$$SR_H^d(t) = \delta\max\left(-\frac{dG(t)}{dt}, 0\right) \tag{S7d}$$

Here H (in ng/L) is the concentration of plasma glucagon; SR_H (in ng/L/min) is the glucagon secretion; Ra_H (in ng/L/min) is the rate of appearance of glucagon in plasma; SR_H^s (in ng/L/min) and SR_H^d (in ng/L/min) is the static and dynamic components of glucagon, respectively; n, ρ, I_{th}, δ are the parameters.

Subcutaneous insulin kinetics:

$$R_{ia}(t) = k_{a1}I_{sc1}(t) + k_{a2}I_{sc2}(t) \tag{S8a}$$

$$\dot{I}_{sc1}(t) = -(k_d + k_{a1})I_{sc1}(t) + IIR(t), \qquad I_{sc1}(0) = I_{sc1ss} \tag{S8b}$$

$$\dot{I}_{sc2}(t) = k_d.I_{sc1}(t) - k_{a2}I_{sc2}(t), \qquad I_{sc2}(0) = I_{sc2ss} \tag{S8c}$$

$$IIR(t) = IIR_b + \frac{u_I(t)}{BW} \tag{S8d}$$

Here R_{ia} (in pmol/kg/min) is the rate of appearance of insulin in plasma; I_{sc1} (in pmol/kg) is the amount of nonmonomeric insulin in the subcutaneous space; I_{sc2} is the amount of monomeric insulin in the subcutaneous space; $IIR(t)$ is the insulin infusion rate where IIR_b is the basal infusion rate (in pmol/kg/min) from body and u_I (in pmol/min) is the external insulin infusion rate; k_{a1}, k_{a2}, k_d are the parameters. As the exogenous insulin infusion rate appears in the above equation in pmol/kg/min, we divide u_I by the body weight BW in the equation. Note that here the u_I is in pmol/min. To convert the unit of insulin infusion rate u_I from U/min to pmol/min, we multiply u_I by 6944.4, that is the unit conversion is 1 U/min = 6944.4 pmol/min.

Subcutaneous glucagon kinetics:

$$\dot{H}_{sc1}(t) = -(k_{h1} + k_{h2})H_{sc1}(t) + GIR(t), \qquad H_{sc1}(0) = H_{sc1ss} \tag{S9a}$$

$$\dot{H}_{sc2}(t) = k_{h1}H_{sc1}(t) - k_{h3}H_{sc2}(t), \qquad H_{sc2}(0) = H_{sc2ss} \tag{S9b}$$

$$Ra_H(t) = k_{h3}H_{sc2}(t) \tag{S9c}$$

$$GIR(t) = GIR_b + \frac{u_G(t)}{BV} \tag{S9d}$$

Here H_{sc1} (in ng/L) and H_{sc2} (in ng/L) are the glucagon concentration in the subcutaneous space; IGR is the glucagon infusion rate where GIR_b is the basal glucagon infusion rate (in ng/L/min) from the body and u_I is the external glucagon infusion rate (in ng/min);k_{h1}, k_{h2}, k_{h3} are the parameters. As the exogenous glucagon infusion rate appears in the above equation in ng/L/min, we divide u_G by the body volume BV in the equation. Note that here the u_G is in ng/min. To convert the unit of glucagon infusion rate from mg/min to ng/min, we multiply u_G by 10^6, that is the unit conversion is 1 mg/min = 10^6 ng/min.

We write the ODEs in Eqs. (S1)-(S9) in the form $\dot{\mathbf{x}}(t) = \mathbf{f}(\mathbf{x}(t), \mathbf{u}(t), \Theta_{Gb})$ where $\mathbf{x} \in \mathbb{R}^{17}$ and t is the physical time (in min). The variable x_1 represents G_p, the mass of glucose in plasma; the variable x_2 represents G_t, the mass of glucose in tissue; the variable x_3 represents the mass of liver insulin I_l; the variable x_4 represents the mass of plasma insulin I_p; the variable x_5 represents the amount of delayed insulin I' in compartment 1; the variable x_6 represents the amount of delayed insulin X^L action on EGP; the variable x_7 represents the amount of solid glucose Q_{sto1} in the stomach; the variable x_8 represents the amount of liquid glucose Q_{sto2} in the stomach; the variable x_9 represents the glucose mass Q_{gut} in the intestine; the variable x_{10} represents the amount of interstitial fluid X; the variable x_{11} represents the amount of static glucagon SR^s_H; the variable x_{12} represents the amount of plasma glucagon H; the variable x_{13} represents the amount of delayed glucagon X^H action on EGP; the variable x_{14} represents the amount of nonmonomeric insulin I_{sc1}; in the subcutaneous space; the variable x_{15} represents the amount of monomeric insulin I_{sc2} in the subcutaneous space; the variable x_{16} represents the amount of subcutaneous glucagon H_{sc1} in the subcutaneous space; the variable x_{17} represents the amount of subcutaneous glucagon H_{sc2} in the subcutaneous space. Also $\mathbf{u}(t) = [u_I(t) \quad u_G(t)]^T$, where u_I is the external insulin and u_G is the external glucagon. We define Θ_{G_b} as the set of parameters for which the basal glucose level is G_b.

Parameters

There are a total of 46 parameters in Eqs. (S1)-(S9). The parameters are not given in [3]. We set all the parameters for 'Glucose subsystem', 'Insulin subsystem', 'Glucose rate of appearance', 'Endogenous glucose production', 'Glucose utilization', 'Glucose utilization', 'Renal excretion', 'Subcutaneous insulin kinetics' from the references [1,2], except k_{p1}, V_{m0} and HE_b. According to [2], the parameters are chosen to satisfy the steady-state constraints in type I diabetes. The parameters k_{p1} and V_{m0} are set so that the steady state solutions provide the basal Glucose level G_b and $EGP_b = 2.4$. In Type I diabetes, the endogenous glucose production is high [2], so we choose $EGP_b = 2.4$ mg/kg/min. We set $IIR_b = 0$ and $GIR_b = 0$ as the model we consider is for Type I diabetes. The commercial version of the UVA/Pavoda simulator [67] allows computing blood glucose responses to supplied dosages of insulin for some patients, but does

not provide all of the parameters. We tune the parameter HE_b so that the blood glucose response to insulin of the *patient* we consider in this paper is qualitatively similar to the blood glucose response to insulin of a patient from the software [67] (adultaverage.mat). All of the parameters we use are listed in the S1 Table for reproducibility of the results. Our implementation of the model [3] has been published in GitHub [68].

The equations for k_{p1} and V_{m0} are given below:

$$k_{p1} = EGP_b + k_{p2}G_{pb} + k_{p3}I_b \tag{S10a}$$

$$V_{m0} = \frac{(EGP_b - F_{cns})(K_{m0} + G_{tb})}{G_{tb}} \tag{S10b}$$

The basal steady states are given below:

$$G_{pb} = G_b.V_g \tag{S11a}$$

$$G_{tb} = \frac{F_{cns} - EGP_b + k_1 G_{pb}}{k_2} \tag{S11b}$$

$$I_{lb} = I_{pb}.\frac{m_2}{m_1 + m_3} \tag{S11c}$$

$$I_{pb} = \frac{IIR_b}{m_2 + m_4 - \frac{m_1 m_2}{m_1 + m_3}} \tag{S11d}$$

$$I_{sc1ss} = \frac{IIR_b}{k_d + k_{a1}} \tag{S11e}$$

$$I_{sc2ss} = \frac{k_d}{k_{a2}} I_{sc1ss} \tag{S11f}$$

$$SR^s_{Hb} = nH_b \tag{S11g}$$

$$H_{sc1ss} = \frac{GIR_b}{k_{h1} + k_{h2}} \tag{S11h}$$

$$H_{sc2ss} = \frac{k_{h1}}{k_{h3}} H_{sc1ss} \tag{S11i}$$

Here, the basal values G_b (in mg/dL), IIR_b (in pmol/kg/min) and GIR_b (in ng/L/min) are settable by the user.
