# Supplementary material for: Optimal regulation of blood glucose level in Type I diabetes using insulin and glucagon
Source: PLoS One. 2019 Mar 20;14(3):e0213665. doi: 10.1371/journal.pone.0213665 (PMC6426249; doi:10.1371/journal.pone.0213665)
Supplement: S2 Appendix — The network representation of the GIGM model. The references [70–85] are appeared in this section. (PDF) [file pone.0213665.s002.pdf]

S2 Appendix: Network Representation of the GIGM Model

In this section we construct a *graph* representation of the GIGM model. A graph $\mathcal{G}(\mathcal{V}, \mathcal{E})$, consists of a set $\mathcal{V} = \{x_i\}$, $i = 1, \ldots, n$ of nodes and a set $\mathcal{E}$ of directed edges, where each edge is identified by an ordered pair $\{x_i, x_j\}$ of nodes $x_i, x_j \in \mathcal{V}$.

Complex networks typically consist of two parts; a set of nodes with their interconnections which represent the topology of the network, and the dynamics which describes the time evolution of the network nodes. The prescribed dynamics of the nodes can be linear or non-linear. We can construct a complex network from a dynamical system, simply by considering a state variable as a node and by drawing a directed edge from a node x_i to another node x_j if x_i appears in the time derivative of x_j. We call *driver nodes*, the nodes which directly connect to the external inputs. We call *target nodes*, the nodes which have prescribed state that must be satisfied at the final time [50].

A network representation of the GIGM model is shown in S1 Fig. Each one of the state variable x_i, $i = 1, \cdots, 17$, in Eqs. (S1)-(S9) is associated with a node (shown as a green circle in the figure). A directed edge (shown as a black arrow in the figure) is drawn from node x_i to node x_j, if the state x_i appears in the time derivative of the state x_j. For instance, as G_t appears on the right hand side of Eq. (S1a), there exists an edge from node G_t to node G_p. In the model in Eqs. (S1)-(S9), u_I and u_G are the external inputs acting on the nodes I_{sc1} and H_{sc1}, respectively. Thus the set of drivers node $\mathcal{D} = \{I_{sc1}, H_{sc1}\}$. In this model as the plasma glucose G_p is the only variable we are trying to affect through the control action, thus the set of target nodes $\mathcal{T} = \{G_p\}$. In the figure, the driver nodes are colored cyan and the target node are colored magenta. As can be seen, the effect of the control inputs on the target node is mediated by the network structure, thus this particular structure plays an important role in our ability to control the network output. While the effect of the network topology has been investigated in the case of linear network dynamics, see e.g. [70-81], the case of nonlinear networks has so far received only limited attention [82-85]. Reference [42] and this paper investigate this problem in the context of two applications of interest to the medical field.
