# Supplementary material for: Optimal regulation of blood glucose level in Type I diabetes using insulin and glucagon
Source: PLoS One. 2019 Mar 20;14(3):e0213665. doi: 10.1371/journal.pone.0213665 (PMC6426249; doi:10.1371/journal.pone.0213665)
Supplement: S3 Appendix — There are some non-differential functions in Eqs (S1)-(S9). We present the smooth approximations of these non-differential functions in Eqs (S12)-(S15). (PDF) [file pone.0213665.s003.pdf]

S3 Appendix: Continuous Approximation of Non-differential Function in ODEs

The optimization algorithms implemented in $\mathcal{PSOPT}$ require the derivatives of the function $\mathbf{f}(\mathbf{x}(t), \mathbf{u}(t), \Theta_{G_b})$ exists. We notice that there are discontinuities in Eqs. (S1)-(S9).
The smooth approximation of the Renal exertion function $E(t)$ in Eq. (S6) by using a Heaviside function is,

$$E(t) = k_{e1}(G_p(t) - k_{e2}) \times \mathcal{H}(G_p(t), k_{e2}, k), \tag{S12}$$

where,

$$\mathcal{H}(G_p(t), k_{e2}, k) = \frac{1}{1 + e^{-k(G_p - k_{e2})}}, k \in \mathbb{Z}. \tag{S13}$$

Here a larger k corresponds to a sharper transition around $G_p(t) = k_{e2}$.
We define a continuous approximation of the Dirac delta function $\delta(t - \tau_D)$ in Eq. (S3c),

$$\delta(t - \tau_D) = \frac{d}{dt}\mathcal{H}(t, \tau_D, k), \tag{S14}$$

where $\mathcal{H}(t, \tau_D, k) = \frac{1}{1+e^{-k(t-\tau_D)}}, k \in \mathbb{Z}$. Here a larger k corresponds to a sharper transition at $t = \tau_D$.
We also define continuous approximation of the $\max(.)$ function, e.g. in Eq. (S4d), as

$$\max(H(t) - H_b, 0) = (H(t) - H_b) \times \mathcal{H}(H(t), H_b, k), \tag{S15}$$

where $\mathcal{H}(H(t), H_b, k) = \frac{1}{1+e^{-k(H-H_b)}}, k \in \mathbb{Z}$. Here a larger k corresponds to a sharper transition at $H(t) = H_b$. In all our approximation we set $k = 4$.
