# Supplementary material for: Optimal regulation of blood glucose level in Type I diabetes using insulin and glucagon
Source: PLoS One. 2019 Mar 20;14(3):e0213665. doi: 10.1371/journal.pone.0213665 (PMC6426249; doi:10.1371/journal.pone.0213665)
Supplement: S1 Table — Average Parameters of the GIGM model. (PDF) [file pone.0213665.s004.pdf]

Table S1 Table. Average parameters

Parameter	Type I Value	Unit
BW	78 [1]	Kg
BV	78 [1]	L
V_g	1.49 [1]	dL/kg
k_1	0.065 [1,2]	min^{-1}
k_2	0.079 [1,2]	min^{-1}
V_I	0.04 [1]	L/kg
m_1	0.379 [1]	min^{-1}
m_2	0.673 [1]	min^{-1}
m_4	0.269 [1]	min^{-1}
m_5	0.0526 [1]	min.kg/pmol
m_6	0.8118 [1]	dimensionless
HE_b	0.112[1]	dimensionless
k_{p1}	change Eq. (**??**)	mg/kg/min
k_{p2}	0.0021 [1,2]	min^{-1}
k_{p3}	0.009 [1,2]	mg/kg/min per pmol/L
k_{p4}	0.0786 [1]	mg/kg/min per pmol/L
k_i	0.0066 [1]	min^{-1}
$k_{\max}$	0.0465 [1]	min^{-1}
$k_{\min}$	0.0076 [1]	min^{-1}
k_{abs}	0.023 [1]	min^{-1}
k_{gri}	0.0465 [1]	min^{-1}
f	0.9 [1]	dimensionless
a	0.00016 [1]	mg^{-1}
b	0.68 [1]	dimensionless
c	0.00023 [1]	mg^{-1}
d	0.009 [1]	dimensionless
F_{cns}	1 [1]	mg/kg/min
V_{m0}	changes (Eq. (S10)	mg/kg/min
V_{mx}	0.034 [1]	mg/kg/min per pmol/L
K_{m0}	4661.21	mg/kg
P_{2u}	0.084 [1]	min^{-1}
k_{e1}	0.0007 [1]	min^{-1}
k_{e2}	269 [1]	mg/kg
k_d	0.0164 [2]	min^{-1}
k_{a1}	0.0018 [2]	min^{-1}
k_{a2}	0.0182 [2]	min^{-1}
δ	0.682 [3]	(ng/L per mg/dL)
σ	1.093 [3]	min^{-1}
n	0.15 [3]	min^{-1}
ζ	0.009 [3]	(mg/kg/min per ng/L)
ρ	0.57 [3]	(ng/L/min per mg/dL)
k_H	0.16 [3]	min^{-1}
I_{th}	I_b [3]	(pmol/L)
G_{th}	G_b [3]	mg/dL
k_{h1}	0.0164 [3]	min^{-1}
k_{h2}	0.0018 [3]	min^{-1}
k_{h3}	0.0182 [3]	min^{-1}
