# Supplementary material for: Optimal regulation of blood glucose level in Type I diabetes using insulin and glucagon
Source: PLoS One. 2019 Mar 20;14(3):e0213665. doi: 10.1371/journal.pone.0213665 (PMC6426249; doi:10.1371/journal.pone.0213665)
Supplement: S2 Table — Basal values of the GIGM model. (PDF) [file pone.0213665.s005.pdf]

Table S2 Table. Basal values

Basal	Type I Value	Unit
X_b^H	0 [69]	pmol/L
EGP_b	2.4 [2]	mg/kg/min
H_b	93 [69]	ng/L
IIR_b	0 [2]	pmol/kg/min
